# Schools as Drivers of Capitalist Accumulation Conditional Socialized Reproduction in Shenzhen

**DOI:** 10.1007/s10624-022-09682-5

**Published:** 2023-01-11

**Authors:** Anne-Christine Trémon

**Affiliations:** Laboratoire d’anthropologie culturelle et sociale, Université de Lausanne, Switzerland, Centre Chine -Corée -Japon, École des Hautes Etudes en Sciences Sociales, Paris, France

**Keywords:** Capitalism, Social reproduction theories, Althusser, Bourdieu, Circuit of capital, Schools, Public goods, Socialized capital, Class, Surplus population, Shenzhen

## Abstract

Based on fieldwork in an urbanized village of Shenzhen, this paper analyzes the place of schools in the reproduction of Chinese state capitalism. It retraces the circuit of socialized capital that allows for the social reproduction of the native elite and the exclusion of many migrant workers in the context of Shenzhen’s development as a special economic zone and its efforts to upgrade the economy. The native villagers, now forming an urban upper-class of rentiers, have capitalized on their overseas connections and capital accumulation to finance their school, allowing for their elite’s upward social mobility after, but also already under Mao. After China’s transition to capitalism, this school has served as an asset in generating value in the context of redevelopment and the real estate-driven upgrading of Shenzhen’s economy. Property ownership is now a major criterion in points-based systems for accessing school places. I make two interrelated arguments. First, there is a closer relationship between the secondary circuit of socialized capital and the larger circuit of capital than what the literature on social reproduction implies. Second, the conditionality of quality education upon value generation amounts to separating the population deemed worthy of socialized reproduction and the surplus population that is left out. The paper connects diverse strands of social reproduction theory, Althusser’s interpellation and ideological state apparatuses, feminist agentive social reproduction theories, and Bourdieu’s capital conversion recuperated within a Marxian framework, to provide an integrated approach to social reproduction within capitalism.

In China, educational inequalities remain high in spite of the central state’s commitment to universalize access to public education.^1^ China’s economic and social development depends on the education of its future workers to limit the slowdown of economic growth and for social cohesion. However, one major contradiction exists between the general aim of heightening the overall “quality” (*suzhi*) of the population and the constraints placed by budgetary scarcity on local governments responsible for educational expenses.^2^ This results in a gap between the principle of equal access to public education and the actual mechanisms of allocation of school places.

This gap is particularly large in Shenzhen, the Chinese city that has the largest share of internal migrant population due to its development as a Special Economic Zone (SEZ). Shenzhen was the largest and the most ambitious of the Special Economic Zones that opened up in China’s southeastern coastal provinces after the beginning of the economic reforms and that spearheaded China’s reforms and transition to state capitalism (Vogel 1989; Sklair 1991; Ong 2004). Chosen because of its vicinity to Hong Kong, the zone comprised several hundred villages whose inhabitants capitalized on their overseas and Hong Kong connections to attract donations for public infrastructure and investments in factories.^3^ Shenzhen’s economy primarily relied on the manufacturing of export goods until it was hit by the global economic crisis in 2008, which caused many factories to shut down. The Chinese authorities reacted by intensifying the upgrading of Shenzhen’s economy toward less labor-intensive high-tech industries, and by fostering real estate-driven growth, urging the redevelopment of its urbanized villages, where blue-collar migrants had found cheap housing and employment in the Hong Kong and Taiwanese owned factories.

Based on a study of the provision of public goods, including schools, in one such former rural village that has become a neighborhood of Shenzhen, this paper analyzes their place in the reproduction of Chinese state capitalism. I provide a historical account of how the local school has become an asset in the village’s redevelopment and of how in turn access to this school, as to schools more generally in China, is tied to real estate assets. The case study presented here shows that the contradiction between, on the one hand, the increasing need for an educated workforce and commitment to equal access to public education and, on the other, the budgetary scarcity resulting from the prioritization of capitalist accumulation is provisionally solved by the indexing of the provision of schools and access to education to the generation of value.

Schools occupy a special place in what Nancy Fraser calls (2016) the “separation-cum-dependence-cum-disavowal” of social reproduction in the functioning of capitalism. Schools (unlike individual private tutoring, or homeschooling) are institutionalized forms of collectively provided social reproduction — “socialized reproduction.” Therefore, they are usually financed through channels of pooled-together contributions or taxes, which are meant (at least in theory) to ensure some amount of redistribution to ensure that all children benefit from public education. Under capitalism, socialized reproduction is financed by a secondary circuit within the circuit of capital.

I make two interrelated theoretical arguments. First, there is a closer relationship between the secondary circuit of socialized capital and the larger circuit of capital than what the literature on social reproduction implies. For Althusser (2011, pp. 77–78), under capitalism, education plays a privileged role by providing the skills for production, but mostly by ensuring the social division of labor. Ideology, which plays a central role in the reproduction of the conditions of production, is relatively autonomous from the productive process. Schools, the material expression of ideological state apparatuses, ensure the distribution of unequal places within the wider social system. For feminist social reproduction theorists Vogel (1983), Bhattacharya (2017a, b), and Ferguson (2020), the space of capitalist production of value and the space of non-capitalistic production of labor power are distinct.^4^ Public schools, like reproductive household activities, belong to the categories of goods and services that have use value but no exchange value (Bhattacharya 2017a, p. 8). Their funding by the state places schools in an external relation with the circuit of capital. Thus, while remaining essential for capital, the production of labor power takes place “outside its immediate circuit” (Bhattacharya 2017b, p. 85).

The circuit of financial provision for education may be separate, but it is not independent — it is shaped directly or indirectly by the circuit of capital. To retrace the circuit of socialized capital, I connect Bourdieu’s principle of convertibility between capitals with Lebowitz’s (2003) and Harvey’s (2017) notion of secondary and tertiary circuits.^5^ Although Bourdieu has been criticized on several grounds to which I return in the course of the article, using his notion of capital conversion, which was central to his writings on social reproduction, allows recuperating part of his theory within a Marxian framework (Burawoy (2021) makes different suggestions for such a recuperation).

I further argue that the indexing, or the conditionality of quality education upon value generation, amounts to separating the population deemed worthy of socialized reproduction and the surplus population that is left out. In the context of China’s state capitalism, the surplus population is not surplus in the sense that it is useless to production in the present, but insofar as it is not viewed as worthy of investing in its children’s future. McIntyre and Nast (2011) and Li (2010) combine Marx’s notion of the “relative surplus population” (the part of population that is surplus to capital, see Marx 1992, Smith 2011) and Foucault’s biopolitics to outline the way racialized surplus populations are left out of governmental interventions aimed at keeping populations alive and ensuring their reproduction. Friedman (2018) argues along the same lines that because large Chinese cities avoid the costs of social reproduction of migrant workers, these constitute a (nonracialized) surplus population.^6^ Though close, my argument is slightly different: The surplus population is excluded, according to a circular logic, from socialized reproduction because of its estimated incapacity to contribute to socialized reproduction. This exclusion reveals a tension, imbedded in the transition from an industrial-export economy to a high-tech and service economy, between the demands of industrial and real estate capital.

I adopt a longitudinal and meso-observational lens, at the level of a rural village turned into an urban neighborhood, using biographical, observational, and historical material I collected over the course of seven stretches of fieldwork in Shenzhen between 2011 and 2018.^7^ The diachronic depth offered by this material allows the examination of the social reproduction of class in this community. The meso-focus on the two classes now cohabiting within the urbanized community, a minority of native villagers (1800 out of a population of almost 60,000 in 2018) and a large majority of migrant workers, brings into relief sharp differences in terms of access to public education.

Retracing the history of the primary school in the Shenzhen urban village of Pine Mansion, I first show that the inhabitants of this village, native Shenzheners, have invested in their school and in education as part of a social ideal of the well-educated subject who is oriented toward the realization of self, but can equally well be interpreted through Althusser’s notions of state ideology and interpellation. While all Chinese citizens desire securing good public education for their children, the link between property ownership and access to schools favors middle-class household strategies. The history of school funding reveals that a circuit of socialized capital has been shaped following mainly overseas capital accumulation. The second section shows that this school has served as an asset in urban redevelopment and the generation of value for developers, the government, and property buyers. In China’s real estate-driven red capitalism, state investment in schools is conditional upon urban redevelopment projects, and more generally, differentially distributed, depending on development priorities. The third section looks at the point-based system for public school admissions, which discriminates between high and low “quality” citizens. Faced with pressure from the central state to grant migrants access to local citizenship and public education for their children, large cities such as Shenzhen have devised selective point systems that prioritize those who hold economic capital, particularly in the form of real estate property. Access to urban citizenship (*hukou*) and to better funded state schools is determined based on age, degrees, and potential contribution to the city budget. In this way, urban governments incorporate some people in the sphere of socialized social reproduction while excluding surplus others.

## The social reproduction of a literate elite before and after Mao

In the post-Mao era, the Chinese state has abandoned the language of class struggle and substituted it with Hu Jintao’s concept of the “harmonious society” and the rhetoric of social strata of higher or lower quality, *suzhi*. The native villagers of Pine Mansion, which form a class of rentiers who have benefited tremendously from the creation of the Shenzhen Special Economic Zone, strongly adhere to this vision.

Pine Mansion was, before it became absorbed in Shenzhen city, an almost single-lineage rural village once chiefly inhabited by members of the same lineage.^8^ The vast majority of its native residents bears the surname Chen and claim common descent from their founding ancestor. In April 2017, on the weekend before Qingming, when visiting relatives congregate with their village kin for ancestral worship and lunch, I met Chen Cuixiao, an elderly woman who was so talkative that our conversation soon turned into an informal interview. She introduced herself by saying she wanted me to talk to her granddaughters, mentioning proudly that one of them was studying in the UK.

Born in 1936, Cuixiao is a retired primary school teacher. She originates from a rich merchant family in the small border town of Shenzhen, which gave its name to the new metropolis that emerged with the creation of the Shenzhen Special Economic Zone in 1980. Cuixiao lives in what has become downtown Shenzhen, but her late husband owned a seven-story building in Pine Mansion village together with his brothers. She and her brothers-in-law stay there when they visit Pine Mansion, renting out the other floors to migrants. She has eight grandchildren, four in Hong Kong, four in Shenzhen, but, she said jokingly, “We all get together harmoniously now, [whereas] in the past we had many ‘classifications’ (*chengfen*).” Under Mao, Chinese rural citizens were classified into different peasant categories by the social class system introduced in the countryside with the land reform (Wu 2014). The system favored medium-rich and poor peasants but discriminated against the landowners and rich peasants in terms of the amount of agricultural work to be done as well as their access to education.

Cuixiao’s husband’s family was classified as belonging to the landlord class. Cuixiao contrasts this Maoist past with the present era in terms of access to education and class differences:

In the past, in China, it was called the “exploiting class”; that is, those who exploited others [giggling]. That’s the meaning. At that time, we were discriminated against in studying. (…) So, many of us went overseas, just to survive, not for any other reason. Fortunately, thanks to Deng Xiaoping, [who] opened up China, we came back very quickly. We were very happy, and we have property. Children are studying in the United States and the United Kingdom, all can support themselves.

Cuixiao emphasizes her satisfaction with a return to a normal state in which all “young people can study.” Parents’ desire to see their children succeed in their studies reposes on Confucian ethics of filiality and cultivation of selfhood (Kipnis 2009, p. 219). However, as Kipnis notes, state and family desires are mutually imbricated (ibid.). The notion that a person achieves self-realization through education is the outcome of an ideology whereby, following Althusser (2011), the state “interpellates” its subjects and social hierarchy is reproduced. The lineage is a form of social organization that is dedicated to realizing both this Confucian personal ethos and this state ideology. “Invented” during the Confucian revival movement of the fifteenth and sixteenth centuries (Faure 2007, p. 219), lineages draw prestige from the numbers of their degree holders and successful candidates in the imperial examinations.

Lineages cultivate and enable some amount of “interclassist” solidarity (Freedman 1958, p. 127; Faure and Siu 1995, p. 6; Unger 2002, p. 37). Prior to 1949, most lineage land was held in trust in the name of the founding ancestor or later ancestors, and part of its income was used to fund ancestral worship, philanthropic works, and lineage schools. In the late imperial and Republican period, many lineages turned their private elite schools into public schools for all, and the Chen Pine Mansion lineage was no exception. Influenced by returnees from overseas, the villagers decided to set up a public primary school in 1914, by pooling overseas donations and local income from the land held in common. Prohibited under Mao, the lineage resurfaced after the reopening. Nowadays, the lineage foundation and the village’s shareholding company, which both belong to the native villagers, perpetuate this redistributive system by collaborating in funding scholarships of up to 10,000 RMB per year for young native Pine Mansioners who succeed in gaining a university place.

Moreover, as Hill Gates and others have emphasized, the merchant class and the capitalist mode of production were kept in check by the Chinese imperial state: Being wealthy was never enough to be considered part of the state elite — economic capital had to be converted into scholarly capital. Cuixiao opposed being poor to being educated: “You know, we overseas Chinese, we weren’t poor, we were literate.” She exposes an ideal that — as Bhattacharya and Ferguson emphasize — partly escapes the logic of capitalist alienation because it is primarily an ideal of self-realization.^9^ However, lineages are marked by strong class inequalities due to the way their members are committed to the accumulation of wealth through both merchant activities and the acquisition of land. It is also clearly the case that the overseas social and economic capital amassed was converted into cultural and educational capital. For instance, Cuixiao’s brother-in-law, a university professor, was able to study thanks to the money sent back from overseas in the 1950s by his uncle, who ran a successful import–export company overseas.

Althusser (2011, p. 180) explicitly leaves aside the reproduction of labor power and emphasizes the autonomy of ideology.^10^ His notion of “interpellation” by state ideology, which molds subjects’ desire to educate themselves to a position within the state system of social stratification, does not give “any autonomy at all to the constitutive agents in, and through whom, the relationship can only be formed” (Willis 1981, p. 52). Bhattacharya and Ferguson’s social reproduction theories, with their emphasis on education as a means of self-realization, are agentive in that they foreground reproductive and productive labor. However, as Gimenez (2019, p. 83 fn 30) argues, they result in elisionism: they conflate structure and agency by reducing everything to action (labor). Both theoretical strands are complementary, in that the first foregrounds structure while the second foregrounds agency. Yet both tend to conflate structure and agency in ways that are highly problematic.

Although Bourdieu’s theory has been critiqued for likewise conflating agency and structure (but see Elder-Vass (2007) for a more nuanced approach), his theory of capital conversion provides one first missing bridge by showing how social agents’ practices of capital accumulation and conversion reproduce class inequality (Bourdieu 1986; Bourdieu and Passeron 1990).Ferguson (2017 unpaged) notes this framework limits the purview of social reproduction to the way class inequality is “reflected” in unequal education and transmitted through school capital “as a ‘thing’ one possesses and not as a relation between the dispossessed and the owners of the means of production.” However, instead of dismissing his theory, we should retrieve his reading, which ultimately rests on an understanding of capital as accumulated and appropriated labor to which every other form of capital is reducible in the last analysis:

Capital is accumulated labor (in its materialized form or its “incorporated,” embodied form) which, when appropriated on a private, i.e., exclusive, basis by agents or groups of agents, enables them to appropriate social energy in the form of reified or living labor (1984, p. 15)

Depending on the field in which it functions (…) capital can present itself in three fundamental guises as economic capital, which is immediately and directly convertible into money and may be institutionalized in the form of property rights; as cultural capital, which is convertible, in certain conditions, into economic capital and may be institutionalized in the form of educational qualifications; and as social capital, made up of social obligations (“connections”), which is convertible, in certain conditions, into economic capital and may be institutionalized in the form of a title of nobility (1984: 16).

Capital is a relationship between classes, inherent to capitalist society, a society whose reproduction depends on the circulation of capital and where those who own value-producing assets exploit the labor of those who do not (see Narotzky and Smith 1986, pp. 108–109; Narotzky 2007, p. 409).

Cuixiao was from a bourgeois urban family, but the fact that she married into the Chen rural-based lineage signals the wealth and social standing achieved by some of its members, more specifically, one of its most powerful segments, the Guobao branch, which is named after an immensely wealthy merchant who lived at the end of the nineteenth century. Thanks to the wealth they inherited from him, many members of this branch were able to study, and even if their property was confiscated during the land reform of the early 1950s, they held enviable urban administrative careers during most of the Mao era. Because of their overseas connections, they were particularly exposed to discrimination and attacks during the Cultural Revolution, which motivated part of them to flee to Hong Kong, and from there, to migrate overseas.^11^ Yet many returned in the 1980s, at the start of China’s reforms, and resumed possession of part of their confiscated property.

As a result, this branch is specifically rich in people who have accumulated overseas social capital and educational capital, as well as recovered part of their economic capital in the form of collective and individual landed property after the reopening. Its members have a high degree of continued solidarity. They meet once a month and even manage their own branch fund, which draws income from real estate.^12^ Now that the village economy has become prosperous thanks to the income drawn from industrial and residential buildings rented out to multinationals and migrant workers from China’s inner provinces, local wealth sponsors overseas education, while they capitalize on the previous generation’s overseas connections in destinations such as the UK and the USA. Cuixiao’s grandchildren are studying abroad in countries where their uncles and aunts have settled, and they receive monetary support from their parents and grandparents in China: Cuixiao’s granddaughter was granted 10,000 RMB per year for her studies in international relations at a UK university, and her travels back to China are covered. At the meeting where I met her grandmother, the senior leader in charge of the fund asked her to produce her student ID card to reimburse her the money for her plane ticket.

Not only do individuals convert their economic capital into cultural capital and then back into economic capital, but also — at the level of the lineage-village community as a whole — economic capital gained overseas was collectively invested in the school, forming a secondary circuit of socialized capital invested in social reproduction, which was later transferred to the state, as I show next.

## Accessing education through property and real estate development

It is no wonder that Cuixiao, like many others in the former village, expressed her satisfaction with Shenzhen and China’s economic development: “Now it is a lot better. It’s not that it wasn’t good before, but everyone was having a hard time sustaining himself or herself. [In the past] we couldn’t just sit and chat like this; now, we don’t have to worry about life anymore and we just want to educate our children. If the grandchild is able to study, then we should save a little and support her.” She added, “Studying overseas costs a lot, but you can sell a house if it’s necessary [to cover overseas university costs].” She holds a class-centric moral discourse that reveals the tight nexus between the capacity to educate oneself and property ownership in China. Cuixiao was able to buy an apartment at a subsidized price in the privatized apartment complexes in Shenzhen. The commodification of state-owned housing as a consequence of the Urban Real Estate Administration Law of 1995 has mainly benefited the urban middle class of state employees (Tomba 2014).

Many upper-class members of the lineage who have led urban careers live in the center of Shenzhen and other surrounding cities such as Guangzhou, while earning rental income from their property in Pine Mansion. Others, among which are returned Hong Kongers, have chosen to live in Pine Mansion, as do local shareholding company leaders, administrative cadres at the village and sub-district level, and private entrepreneurs. They prefer living there because they have larger houses than are available in downtown Shenzhen and in Hong Kong. Another reason for preferring Pine Manson is its highly reputed schools.

Some China scholars have started shifting attention to what they call *jiaoyufication*, or education-led gentrification (the term combines the Chinese word for education, *jiaoyu*, and “gentrification”). Several factors account for this process: the change in school zoning policy, the commodification of housing, and the competition for unequally endowed public schools. The 1996 Compulsory Education Law, which made 9 years of education compulsory nationwide, linked locality and education provision by introducing a catchment zone policy for school enrollment. Within a school catchment area, each legal housing unit has a school place guaranteed for 9 years. Meanwhile, the commodification of housing from the mid-1990s onwards has meant that mainly middle-class urbanites have been able to purchase housing within these catchment zones. At the same time, financing and administration of schools were decentralized while fiscal resources were recentralized. Considerable disparities in educational provision reflect local governments’ revenue.

Not only do inner-city urban governments generally have greater means to fund schools, they also inherit better funded schools. This is a legacy of the Maoist dichotomy between rural and urban public goods regimes, and also of the privilege in terms of resources accorded to key schools that were to educate the Party elite (Wu et al. 2016, pp. 3515–3516). Now, the competitiveness of the Chinese educational system fuels this phenomenon of distinguishing better quality public schools, although key schools were banned by the revised Compulsory Education Law in 2006 (and tuition fees were abolished in all primary and middle public schools in 2008). Since the restoration in 1977 of the *gaokao*, a nationwide examination whereby students compete to enter the country’s top universities, students face pressure to enter a top high school through the Grade 9 examination (*zhongkao*). There is no examination to enter a middle school. Being a registered resident of a top primary school’s catchment area makes it easier to enter a top middle school and then a top high school.

Focusing on inner-city neighborhoods, several studies (Wu et al. 2016, 2017; Wu et al. 2018) have pointed out that following the diversification of the elite, which still includes the urban administrative and Party elite, but now includes the business world and professionals, middle-class buyers of privatized housing blocks have displaced an earlier generation of mainly working-class residents in urban areas where former key schools have retained their reputations and resources (Wu et al. 2016, pp. 3515–3516).^13^ To access such top educational institutions, gentrifying parents generally buy a suitably located but often worn-out apartment at an inflated price and sell it after 9 years (covering the period of elementary and middle schooling) to a new gentrifying family. They do not have to upgrade the apartment because its value lies in the school place associated with it.

Therefore, these studies argue that this gentrification process is education-led rather than property-led. Invoking Bourdieu’s distinction between cultural and economic capitals, they tend to overlook his insistence on convertibility between capitals defined as the “guises” capital in the singular takes (see the previous section). Not only is economic capital (institutionalized in the form of property rights) converted into educational capital (degrees) by way of access to a school place, with greater chances of obtaining a place when one owns property (I will come back to this in the next section). Urban redevelopment, a ubiquitous phenomenon in China, bears even more similarity to classic gentrification than the strategies described by the above-quoted studies. All urban local governments in China are enticed to launch urban redevelopment projects to increase their tax bases as a result of the discrepancy caused by the recentralization of fiscal revenue along with the decentralization of financial responsibility for public goods including education (Oi and Zhao 2007; Wong 2010; Jia et al. 2014). Those urban governments that can capitalize on existing prestigious schools are in a better position to attract developers. Real estate companies compete to develop new residential buildings within the catchment areas of well-known schools because they can sell them at much higher prices than in other areas.

Urban villages generally host large sections of migrant populations because they provide inexpensive housing for families. They are also the primary targets of redevelopment policies. Pine Mansion’s primary and middle schools have excellent reputations and they have made the former village very attractive for real estate companies’ investments, as well as for state funding. Pine Mansion’s primary school, established by the Pine Mansion Chen lineage community in 1914, was upgraded using income from several rounds of private, local, and overseas fundraising before 1949 and after China’s reopening. The last round of fundraising took place in 1997, when the people of Pine Mansion mobilized to protest the school’s planned closure and merger with a nearby school. In just a few months, they collected over 2 million RMB. It was only after the old building had been demolished, the new school building finished, and the merger cancelled that the Shenzhen municipal government and two state-owned enterprises granted the project almost a million and 700,000 RMB, respectively. These funds were used to equip the new multistory building with multimedia teaching rooms, a library, a large dormitory, and sports facilities.

Private investment allowed the district government to compensate for its initial lack of resources, but once the city government granted money, it continued doing so. The primary school moved up the ranking from a local school in 2000 to a municipal one in 2003. Shortly after the village was administratively urbanized (it became part of Shenzhen) in 2004, the school became entirely government-funded, which allowed it to reach the highest level as a provincial school in 2005. In Guangdong province, schools are ranked as local, municipal, or provincial according to their size and the quality of their infrastructure. Their ranking determines their funding, which varies according to the level of government providing it. Provincial schools receive local, municipal, and provincial funding, and are therefore the best-resourced and most prestigious.

Although an increasing proportion of Shenzhen city’s expenses is devoted to education, the number of public primary schools has remained stable over the past three decades despite population growth. This is due to the municipality’s preference for subsidizing private primary schools (Wang 2016) and its policy of merging and expanding public primary schools.^14^ The recent expansion of Pine Mansion’s primary school bears a close relationship to Pine Mansion’s urban redevelopment. The school was one of thirty-five being remodeled in Shenzhen in 2018, all in redeveloping communities. The district government has spent 58 million RMB on doubling the school’s surface area and increasing the number of classrooms from twenty-six to sixty. On its completion in 2020, Pine Mansion’s expanded school had places for an additional 1530 students.

Significantly, this extension was decided only after the local, formerly village-level leaders had signed the redevelopment contract in 2011; construction of the new buildings began when the first phase of village renovation was almost complete. The developer used the proximity to and expansion of the provincial primary school in sales literature to illustrate the attractiveness of the future neighborhood. An employee at the sales bureau explained that *shequ fang*—apartments resulting from redevelopment projects—are attractive because they come with a public school place. Buyers thus secure public education for their child.

The city government thus indexes the provision of public goods (in this case, education) and its public expenditures on economic growth, principally by generating value derived from real estate. Bhattacharya notes that the case of schools challenges the spatial divide between capitalist production in the workplace and non-capitalist reproduction at home. However, schools challenge considerations in terms of spatial functionalities *tout court* when considered from the point of view of the total circuit of capital. While they certainly represent use value in the eyes of those who are able to access them in order to educate their children, schools also represent exchange value in terms of the increased future value of apartments in the neighborhood to property buyers.

About one-quarter of the buyers were middle-class native villagers who had the means to borrow the money needed to buy the extra square meters, in addition to the square meters they received in compensation for the demolition of their old houses. The rest of the buyers were middle-class couples from the core urban districts in Shenzhen, where the price per square meter had reached such heights that housing had become unaffordable: Shenzhen has the highest housing prices per square meter in China. Such redevelopment projects also end up displacing working-class people who cannot afford to buy an apartment in the new residential complexes, or who can no longer afford rent.

## Surplus populations do not score high enough: point-based systems for accessing education

In 2017, Pine Mansion’s primary school had over 900 students, 10% of whom were the children of native villagers (who represent a little less than 10% of the population). The rest were the children of new residents with temporary permits. The apartments were to be delivered in the summer of 2018, just after my last stay. The new school places were attached to the new apartments. While awaiting the demolition of the remaining sections of Pine Mansion as part of Phases 2 and 3 (from 2022 to 2036) of the redevelopment project, migrant families continued to rent apartments and send their children to the public primary school. However, many met with increasing difficulties in getting school places.

The Compulsory Education Law, revised in 2006, reaffirmed the central government’s “two primaries” policy, initiated in 2001, which made host localities financially responsible for the education of migrant children in public schools. However, because of the financial burden this represents for localities with high numbers of migrants, they adopt restrictive measures that exclude a high proportion of migrant children from public education. Until recently, the *hukou* system performed this exclusion. By denying migrants welfare benefits and many social services in the localities where they live and work, this system has devolved the costs of social reproduction upon the migrants and the localities from which they originate (Friedman 2018; Chuang 2020).

Due to its specific history of almost ex nihilo creation and emergence as a special economic zone, by 2015, Shenzhen had a population of almost 15 million, of which 76.2% had temporary resident permits and only 23.7% were permanent urban *hukou* holders (Shenzhen Statistics Bureau 2016). Leslie Sklair (1991) described Shenzhen’s growth as resting on a process of “temporary urbanization” whereby temporary permits are issued to migrants who can theoretically be sent home at any time. Low-skilled migrants are indeed being driven away. Starting from the late 1990s and intensifying after the 2008 global economic crisis, Shenzhen has actively sought to upgrade its economy by encouraging investments in higher-tech, greener, and less labor-consuming industries and services. Urban redevelopment projects are part and parcel of such policy, since they entail demolishing entire urban villages, including not only houses but also first-generation factories.

In 2012, Shenzhen introduced a points system facilitating the acquisition of a permanent city *hukou*, but only for select applicants (Zhang 2012). If they meet the qualification criteria, applicants are granted a *hukou* transfer based on the available quota. Such quotas, which vary annually, allow the municipal authorities to fine-tune the number of new Shenzhen citizens according to its state of finances, ensuring that the increase in the registered population will not result in greater pressure on the future local budget. It also makes urban citizenship conditional upon past contributions to the municipal economy, since it grants urban *hukou* to high-income migrants who have launched businesses and purchased property. Points are scored based on age, level of education, amount of capital invested in business, and whether property has been purchased.

Since 2014, a points-based management scheme similar to the system for accessing urban *hukou* has regulated applications for school places equally for both *hukou* and non-*hukou* holders. Schools rank applicants based on their numerical scores and admit students in decreasing order, according to the number of available places. Points systems play a pivotal role in population management and resource allocation (Wang and Liu 2018; Dong and Goodburn  2020) they are an instrument for governing the population by making it self-governing. Shenzhen’s points systems are ideologically justified by their aim of “enhancing migrant workers’ sense of belonging, making migrant workers hopeful, hardworking, more law-abiding, and more caring about the city.”^15^ The city authorities see points systems as an incentive for migrant workers to register and plan their futures in terms of places of work and residence.

The points system for allocation of public school places is admittedly transparent and puts an end to corruption and favoritism by school heads; however, although it is supposed to facilitate access to public schools for temporary residents, it has made doing so harder for many. It is the opposite of redistribution, since it distributes public goods to those who accumulate capital.^16^ Indeed, by giving much more weight than before to property and contributions to the city budget, the allocation of school places has become more selective. The criteria for admission that had prevailed before already excluded many poorer, informally employed migrants (Cheng, Nielsen and Smith, 2014), but were at least predictable: You either met them or did not. Shenzhen’s public schools were accessible to all children provided their parents produced a property or rental certificate, a work contract or business license in the desired school’s catchment area, and a certificate of compliance with the single-child policy. In Pine Mansion, those without a formal work contract—people employed in the small, unregistered workshops to which the larger electronics and plastics factories in the area were subcontracting, or informal petty traders—were unable to send their children to school locally.

The problem of securing a formal rental certificate was also widespread, because the poorer migrants often found cheap housing in urban villages, where informal accommodation prevails (Logan et al. 2009; Zhan 2018). Pine Mansion’s grid system (*wangge*) head, in charge of formalizing the housing market and collecting information on the “floating population,” told me in 2018 that school places are a major cause of dispute between native landlords and migrant tenants. Conflicts erupt when tenants need a formal lease to secure a school place, and landlords refuse to provide one because they want to avoid paying taxes on rental income. Property income tax must be paid in order for a property or lease certificate to be issued. This tax, which ranged between 4 and 6% in 2018, is paid on a voluntary basis.^17^ Even when a lease is signed, it may turn out that the school place allocated to a given apartment or house is not available, for instance, when landlords have provided friends in need of a school place with a fake tenancy contract, depriving the official tenant of their legal school place. The establishment of the grid management unit (Wangge) in urban villages is part of an effort by the Shenzhen authorities to reduce informality and social conflict.

However, the shortage of public school places remains acute and is compounded by formalization of housing. The points-based admission system is explicitly presented as a way of allocating this scarce resource. While under the previous system, property contracts and formal rental certificates had the same weight, in the new system, those with local *hukou* or property in the catchment area receive the highest scores and are placed in the top category—that is, the highest priority for school places. The rest are ranked in six further categories with declining chances of getting a place. Points are scored according to whether property is owned in other parts of Shenzhen (owning a house earns more points than renting one), as well as based on the number of years an applicant has contributed to the municipally run social insurance system, or—until 2017—to the number of years they have held a business license. Since 2018, this last item has disappeared from the score sheet: Only contributions to social insurance earn points.

When we met, Gong Zhihui had just realized that for this reason, she would not be able to get her son into the local public middle school next year. Her two sons were then at the local public primary and middle schools, and in 2018, her youngest son was due to move up to middle school. Together with her husband, she had run a shop in Pine Mansion since 2006, so she scored very highly until that item was dropped. Her insurance score—they had started buying insurance only when the points system started in 2014—was not enough to make up for her lack of local property. “Afterward, […] basically we were not able to keep up [*gen bu shangle*]. Many people bought an apartment; they are classified as *diwulei* [Grade 5].” Her own score was at the bottom of Grade 6, and she no longer had enough points to get her second son into the public middle school. She had no other option than to enroll her son at the less reputed and more expensive (although the Shenzhen municipality subsidizes school fees) private school.

While Zhihui did not have the means to buy an apartment in Pine Mansion, she did not complain about those who jump the queue by buying property. Instead, she blamed the choice that she and her husband had made at the very beginning to buy a house in their native village rather than saving for property in Shenzhen. She deplored their own traditionalism: “We built a house in the village. We are traditional over there. [You] have to make a house in your home place first, then elsewhere.” This points to the difficult dilemma in which many migrant workers are caught, juggling between keeping land as a safety net in their home village and building a house on it, or renouncing all rights in their place of origin by selling the land and investing as much as possible in, for instance, insurance and property, which might qualify for points and accelerate their *hukou* transfer. Indeed, upon gaining urban citizenship in a large city, successful *hukou* applicants are asked to give up their land-use rights in their native villages or towns.^18^ The quality of welfare benefits and education is better in large cities such as Shenzhen, but since many migrants are engaged in low-paid jobs or risky petty entrepreneurial ventures, land is considered a form of social security (Cai 2016; Tyner and Ren 2016).

Zhihui blamed herself for not making the decision to buy insurance earlier, although she probably did not have the means at the time and could not possibly have anticipated the change in policy. She had been a volunteer at the primary school for several years in addition to her job and childcare responsibilities, and is quite representative of aspiring middle-class women in the way she explained her volunteering activities by stressing the duty to elevate one’s own quality (*suzhi*) and that of her children. In spite of their difference in age and class, she shared with Chen Cuixiao the notion, and gendered imperative, that investing in the education of one’s children is a matter of self-responsibility as much as a contribution to society. She said she fully understood why first-tier cities like Shenzhen have established selective systems for accessing public schools; the quality of educational services provided would be low if they accepted everyone, like schools do in her hometown. The individualization of responsibility and the discourse on quality, which hierarchizes categories of population and places (first-tier cities over other cities, and the city over the countryside), has replaced the language of class struggle in China. This occultation of class domination by the rhetoric of social strata is perhaps one of the best signs that China is a capitalist society.^19^

The point system for accessing school places can be seen as a perfect synthesis of social reproduction theories: it is an ideologically justified technology of governance that channels individual aspirations, but also shapes people’s behavior by making them perform like Bourdieusian social agents in converting economic capital into educational capital; but is also a tool for allocating resources and adjusting socialized capital to the wider circuit of capital.

## Conclusion

The processes described in this paper are the result of the contradictions faced by the Chinese state in combining capitalist accumulation of wealth and socialist commitments. Granting equal education opportunities is essential to uphold the fiction of the “harmonious society” devoid of class struggle, and to maintain social stability by meeting citizens’ aspirations of self-realization through education and keeping alive their faith in the Communist Party’s ability to foster the people’s well-being. In this respect, both Althusser’s emphasis on the role of the educational system as a state ideological apparatus in the reproduction of social formations and feminist theorists’ insistence on reproductive activity, which to some extent escapes capitalist alienation, appear to be mutually complementary and equally valid. A focus on the practices of capital conversion connects social reproduction theories and allows surmounting the dilemma of Althusser’s favoring of structure over agency and feminist theories’ privileging of agency versus structure. Reintegrating Bourdieu’s approach to the social reproduction of class within the wider framework of social reproduction theory requires treating access to good education not just as a factor in the generational transmission of individualized inequality, but as relation between the dispossessed and the owners of capital.

Bourdieu’s framework does, overall, paint a static picture of society as a set of fields, each of them structured by the distribution of capitals treated as if they were individual endowments. I disagree with this second part of her critique: Bourdieu does emphasize a synchronic vision of society as structured by the distribution of capitals. As Burawoy (2021, unpaged) underlines, Bourdieu failed to provide a theory that connects the separate hierarchically structured fields into a totality — a capitalist totality. Retrieving his definition of capitals as different guises of capital and situating practices of capital conversion within the wider circuit of capital makes possible an integrated approach to social reproduction within capitalism as a totality. My historical account of the local school in a former emigrant of Shenzhen, which has become a neighborhood hosting a large immigrant population, has shown how its native villagers, now forming an urban upper-class of rentiers, have capitalized on their overseas connections and capital accumulation to finance their own school, allowing for their elite’s upward social mobility even under Mao, and how this school has served as an asset in generating value in the context of redevelopment and the real estate-driven upgrading of Shenzhen’s economy.

China’s contradictions are a local variant of a more general contradiction of capitalism, which transpires in the ambivalent relationship between capital and social reproduction, one of both dependence and rejection (Fraser 2016), or in the rival goals of reproducing capital and reproducing population (Li 2010). This is reflected in the way in which rather than being independent from the logic of capital accumulation (forming a parallel circuit) socialized capital invested in education is at once crucial to capital accumulation itself (producing the future labor power, but also generating real estate value) and subordinate to it. In Chinese cities more generally, the provision of public schools and access to education are indexed on the generation of value: not only are public schools used as assets in urban development projects and fuel gentrification, school places are distributed in order of priority to those who contribute to capitalist urban growth by buying property. Public education is accessed in China according to a circular logic, at once material and ideological, which traces the contours of the circuit of capital. Public education is made accessible to those who contribute to paying for local public schools by way of their contributions to the municipal budget, which itself depends on capital accumulation. Those who are unable to contribute are excluded and blamed for their lack of *suzhi* (quality); there is little socialized reproduction.

The crudeness of this deliberate conditionality seems to lend support to the structural-functionalist interpretations that Lebowitz castigates under the heading “one-sided Marxism,” which “espouses the view of capital: if the workday declines, it is because capital needs workers to rest (…); if a public school system is introduced, it is because capital requires better- educated workers.” (Lebowitz, pp. 137–138.) However, as Willis (1981, p. 60) remarked, capital would rather “freeze” teenagers than pay for an educational system that has no utility in respect to the low-skilled jobs that await many of them at the end of compulsory education. The demands of capital are in themselves contradictory, reflecting the contradictions inherent to capitalist accumulation.

According to Bourdieu (1986, p. 19), the dominated classes “do not have the economic and cultural means for prolonging their children’s education beyond the minimum necessary for the reproduction of the labor-power least valorized at a given moment.” This last part of the sentence, “at any given moment,” paradoxically points to his theory’s major weakness, namely his static, structuralist insistence on synchronically analyzing society as a set of structured fields, but it also opens space for identifying a diachronic tension in the unfolding of China’s state capitalism.

At the moment when China is seeking to push back the limits of industrial capitalist accumulation by moving to an economy resting on domestic demand and higher added value, it needs a better educated workforce. This explains why cities such as Shenzhen have set up points systems for granting urban citizenship (*hukou*) and school places to future highly educated, high-income earners, while driving out the previous generation of blue-collar workers. China’s population must be educated to upgrade its economy, making it less dependent upon export manufacturing and more firmly based on high-tech and tertiary services, as well as domestic consumption. The contradiction between the principle of equal education and the prioritization of growth has been resolved until recently by making access to public schools conditional upon investments in real estate. The current deflation and threatening explosion of China’s real estate bubble will raise new challenges.

## Footnotes

1 China’s level of inequalities is among the world’s highest (Kanbur, Wang and Zhang 2017; Picketty, Li and Zuckman 2017; Jain-Chandra et al. 2018). Among the main drivers of China’s inequalities in income are difference in access to education and welfare between rural and urban areas as well as between urban citizens and migrant workers holding temporary permits in the cities where they live (Kwong 2004; Liang and Chen 2010; Liu et al. 2009; Goodburn 2009, 2015; Solinger 2018). Many migrants leave their children behind to study in their hometowns. In 2015, there were 18.9 million children studying in the cities where their parents have migrated and 15.5 million left-behind children in 2017 (Hugaiguancha blog 2018).

2 *Suzhi* is a mix of cultural and educational, economic, and moral qualities, referring to both individuals and whole populations; it broadly indicates well-educated, law-abiding citizens in stable employment.

3 On the importance of rural villages in the creation of Shenzhen, see O’Donnell, Wong and Bach (2017) and Du (2020), and on the history of migration and urbanization of emigrant villages, see Trémon (2023). Part of these villages, initially located outside the municipality and SEZ, joined it in the 1990s.

4 Drawing on Lebowitz’s (2003) demonstration of how the production of labor power is essential for capital, these theorists have broadened the scope of social reproduction (and of oppression) by making labor, instead of capital, central (Bhattacharya 2017a, pp. 6–7) — although Lebowitz maintains the circuit of capital is the totality (and see critiques in Munro 2019; Weiss 2021).

5 Harvey (2017) distinguishes between a secondary and a tertiary circuit of capital that represent investments in fixed capital mediated by state power. The secondary circuit, which includes infrastructure such as railways and highways, serves capital much more directly than does the tertiary circuit, which is made of social expenditures on the education and training of labor. I find it hard to maintain this distinction on the basis of what I show in this paper in terms of how socialized capital serves the purpose of and is derived from capital accumulation.

6 *Suzhi* discourse tends to have naturalizing implications, but is not justified or legitimized by racialized categories.

7 For a full account of my fieldwork and my positionality, see Trémon (2022).

8 Lineages claim descent from a joint patrilineal ancestor and form corporate entities, regularly coming together for activities such as ancestor worship. In Guangdong, from which many migrants departed over several centuries, many rural villages were structured into lineages that acted as “emigration agencies” (Watson 1975, p. 82) and channeled funding from overseas.

9 Bhattacharya (2017a, b) and Ferguson’s (2017, 2020) social reproduction theory (SRT) emphasizes the fact that reproductive labor partly escapes the logic of capitalist alienation and may even run counter this logic. They eschew functionalist explanations of services such as education in terms of their utility to capital. On the contrary, in consuming use values such as education, people do not simply reproduce themselves as workers; they also realize themselves as full human beings (Ferguson 2020).

10 See Brohm (1999) for a critique of Althusser’s contradictions.

11 During the Cultural Revolution (1966–1976), the “intellectuals” — all those with a secondary education — and in the countryside, “bad class” villagers — landowners, rich peasants, and particularly people with overseas ties — were the target of attacks and public criticism. In Pine Mansion, several persons had been imprisoned or publicly beaten to death. The class system fell into disuse during the 1980s rather than being officially repealed.

12 Built on the plot of land where the Guobao school used to be. The Guobao branch was one of the first to establish a private school, and this branch school building became the village’s first primary school in 1914, before it was relocated in a new building after a new round of overseas donations in 1929.

13 In general, public schools are more prestigious than private schools are in China. Some international schools are the main exception.

14 The number of primary schools grew from 226 in 1979 to 378 in 2004 and has declined since then, to 337 in 2016. The number of students enrolled in primary schools grew from 526,419 in 2004 to 910,974 in 2016.

15 Shenzhen Municipal Government, 2016. “Several Opinions of the Shenzhen Municipal People’s Government on Further Strengthening and Perfecting Population Service Management.” http://www.sz.gov.cn/zfgb/2016/gb968/201608/t20160823_4316510.htm

16 This can be generalized to all public transfers. Wang (2018) shows that the more privileged members of society continue to benefit more from public transfers in healthcare and pensions as well as education.

17 This may change. In early November 2022, Shenzhen was nominated a pilot area for China’s property tax reform.

18 A new policy is about to be adopted at the time of writing.

19 Chinese citizens do use notions of class and discrimination against migrants when they critique points systems, but space does not allow to deal with this in this article. See Trémon (forthcoming 2023).
